# Genetically modified rice in Iran: scientific progress, policy constraints, and prospects for sustainable food security

**DOI:** 10.1080/21645698.2026.2707837

**Published:** 2026-07-22

**Authors:** Ghaffar Kiani

**Affiliations:** Department of Plant Breeding, Sari Agricultural Sciences and Natural Resources University, Sari, Iran

**Keywords:** Biosafety regulation, Bt genes, critical review, food security, genetically modified rice, Iran

## Abstract

Iran’s rice security faces acute threats from drought, salinity, and pests. A sustained national research program has developed genetically modified (GM) rice to address these challenges. This critical review synthesizes over 15 years of research, applying a systematic literature search and thematic analysis to map the trajectory from gene discovery to field trials. Iranian scientists have demonstrated stable gene integration, successful trait pyramiding, and validated the superior field performance of *Bt* rice lines, including yield increases and reduced pesticide use with a lower environmental footprint. Despite this readiness, a stark innovation-implementation gap persists due to an inoperative regulatory framework and public skepticism. Contrasting Iran’s path with successful models like Bangladesh highlights that scientific achievement alone is insufficient. This study concludes that transforming this potential into tangible benefits for food sovereignty requires enacting functional biosafety regulations and proactive public engagement that reframes domestic GM crops as tools for sustainable intensification.

## Introduction

Rice (*Oryza sativa* L.) is the staple food for more than half the world’s population and is central to food security, rural livelihoods, and cultural identity in Iran.^1^ Iran hosts important centers of diversity for fruit trees and crop wild relatives.^2^ Global rice production must increase by ~25% by 2050 to meet demand, a challenge exacerbated by climate change, dwindling water resources, and land degradation.^3^ Iran exemplifies this crisis: its critical northern rice-growing regions face severe pressures from drought, soil salinity, and pervasive pest infestations, notably by the striped stem borer (*Chilo suppressalis*), causing annual yield losses of 20–30%.^4,5^ Economic assessments have quantified yield losses from stem borer infestations in northern provinces.^6^ While conventional breeding remains essential, its pace–typically requiring 8–12 years for cultivar release–can be inadequate when addressing rapidly evolving pest populations or emergent abiotic stresses; comparative studies in rice have shown that genetic engineering can reduce this timeline by approximately 3–5 years for single-gene traits.^7^ Iran’s experience with crop improvement extends beyond rice to major cereals such as wheat.^8^

Genetic engineering (GE) emerged in the late 20th century as a precise tool for crop improvement. As of 2019 (the most recent year with comprehensive global data), GM crops were cultivated on over 190 million hectares across 29 countries, delivering documented benefits including a 37% reduction in pesticide use, a 22% increase in farmer profits, and a 0.75 billion kg reduction in CO_2_ emissions.^9,10^ However, adoption is heterogeneous, deeply influenced by national regulatory architectures, public perception, and geopolitical considerations.^11^

Within this global landscape, Iran presents a pivotal and under-examined case study. For nearly two decades, a concerted public-sector research program has developed GM rice varieties tailored to local agronomic challenges. This program has achieved significant scientific milestones, producing lines that are genetically characterized and agronomically validated through confined field trials (CFTs). Yet a stark disconnect persists: Iran has a fully developed scientific portfolio for GM rice but remains a non-cultivator.

This review aims to: (1) comprehensively chronicle and analyze the scientific achievements of Iran’s GM rice research; (2) diagnose the multifaceted regulatory, political, and social barriers creating the current impasse; and (3) propose a constructive pathway forward, drawing lessons from comparative global experiences to inform science policy in Iran and similar developing economies.

## Methodology

To ensure transparency and replicability, the methodology of this review is explicitly reported below.

*Review Type and Rationale*: A critical review was selected because the primary objective was not to aggregate all available evidence quantitatively (meta-analysis) or map the breadth of a field (scoping review), but rather to synthesize, evaluate, and critique the existing body of research on GM rice in Iran, identify conceptual and methodological weaknesses in the literature, and develop a theoretically informed explanation for the observed innovation-implementation gap. Handbook references provide standardized approaches for agricultural biotechnology assessment.^12^

Critical appraisal of individual studies was conducted using adapted criteria. Risk analysis frameworks for GM foods should adhere to international standards established by Codex Alimentarius.^13^

*Search Strategy and Sources*: A structured literature search was conducted using the following databases:

*English-language databases*: Web of Science Core Collection, Scopus, PubMed, and Google Scholar

*Persian-language databases*: IranDoc (ganj.irandoc.ac.ir), SID (Scientific Information Database, www.sid.ir), and Magiran (www.magiran.com)

*Regional databases*: CAB Abstracts (for South Asian rice research)

The search strategy combined keywords using Boolean operators:

Population/Context: (Iran* OR “Islamic Republic of Iran”) AND (rice OR *Oryza sativa*)

Intervention/Topic: (“genetically modified” OR “transgenic” OR “GM” OR “*Bt*” OR “*cry1Ab*” OR “biotechnology”) AND (“biosafety” OR “regulation” OR “field trial” OR “LCA” OR “life cycle assessment”)

*Outcomes*: (“yield” OR “pest resistance” OR “environmental impact” OR “public perception” OR “policy”)

No date restrictions were applied to capture the full historical trajectory of GM rice research in Iran (earliest relevant publication identified: 2000). Only peer-reviewed journal articles, official government documents (e.g., National Biosafety Law), and book chapters were included. Conference abstracts, preprints, and non-academic media reports were excluded from the core evidence synthesis, though media reports were used contextually in the social dimension section.

*Inclusion and Exclusion Criteria*: Articles were included if they met all of the following criteria:

*Relevance*: Directly addressed GM rice research, regulation, or public perception in Iran.

*Study design*: Original research (field trials, molecular characterization, LCA, surveys) or policy analysis.

*Language*: English or Persian.

*Publication status*: Peer-reviewed (for empirical studies).

Articles were excluded if they:

Focused exclusively on non-rice GM crops without reference to rice.

Were limited to conventional breeding or marker-assisted selection without a transgenic component Were editorials, opinion pieces, or news items without original data or analysis.

*Data Extraction and Thematic Analysis*: A thematic analysis approach was employed.

Phase application to this review

1. Familiarization: All retrieved articles (*n* = 78 after deduplication) were read in full; key passages highlighted
2. Initial coding: Open coding of findings, methods, and conclusions using NVivo 14
3. Theme search: Codes grouped into candidate themes (e.g., “gene stability evidence,” “regulatory barriers,” “public skepticism”)
4. Theme review: Themes checked against original articles for consistency; ambiguous themes merged or discarded
5. Theme definition: Final themes defined: (a) scientific pipeline phases, (b) regulatory vacuum, (c) social perception, (d) comparative policy models
6. Writing: Synthesis organized according to the four final themes, with critical commentary on evidence quality and gaps

Critical appraisal of individual studies was conducted using adapted criteria:

*For molecular studies*: presence of appropriate controls, replication, and statistical reporting.

*For field trials*: multi-location design, replication, randomization, and biosafety compliance *For LCA studies*: adherence to ISO 14040/14044 standards and clarity on system boundaries and functional units.

For surveys: sample size, sampling method, and response rate.

*Limitations of the Review Methodology*: Several methodological limitations should be acknowledged. First, as a single-author critical review, no independent second reviewer was available for study selection or coding, which may introduce selection bias. Second, gray literature (e.g., unpublished field trial reports from NIGEB) was not accessible, potentially omitting relevant negative or null findings. Third, quantitative synthesis was not attempted due to heterogeneity in outcome measures across studies. Fourth, language limitations restricted Persian-language searches to databases with limited indexing depth. Fifth, the review does not include a formal risk-of-bias assessment for each included study, which would be required for a systematic review but is beyond the scope of a critical narrative synthesis.

## The Iranian GM Rice Research Pipeline

Iran’s GM rice research represents sustained public investment in strategic science. Led primarily by the National Institute of Genetic Engineering and Biotechnology (NIGEB), Sari Agricultural Sciences and Natural Resources University (SANRU), and the Rice Research Institute of Iran (RRII), the program has followed a phased approach. Below, we synthesize the evidence by phase, noting where methodological details from original studies support or qualify the findings (Table 1).

**Table 1.** Progression of transgenic rice research and key evidence in Iran.

Research Phase	Gene / Trait	Cultivar(s)	Key Evidence from Original Studies	Reference	Methodological Notes / Limitations
Gene Stability	*cry1Ab*	Tarom Molaii, Neda, Nemat	Single-copy integration confirmed via Southern blot; Mendelian segregation (3:1) in T2 generation	Kiani et al.^14^	Stability evaluated across two generations only; multi-generational field data not reported
Trait Pyramiding	*cry1Ab + fgr*	Neda, Nemat	Successful pyramiding confirmed by PCR and progeny testing; fragrance retained	Kiani et al.^15^	Controlled environment only; no CFT data for stacked traits
Agronomic CFT	*cry1Ab*	Khazar-derived lines (KHT2, KHT3, KHT4)	Complete control of stem borer (0% white heads vs. 15–20% in controls); yield increase of 18–25%	Dastan et al.^16^	Single-season (2016); multi-year data not available; resistance management strategy not specified
Environmental CFT	*cry1Ab*	Khazar-derived lines	LCA: ~25% reduction in energy use, ~20% reduction in GWP, reduced aquatic ecotoxicity	Dastan et al.^17^	System boundary: cradle-to-farm gate; functional unit: 1 ton of paddy rice; modeled from CFT data, not commercial farms

### Phase I: Gene Discovery, Transformation, and Molecular Characterization (2000s)

The initial phase focused on establishing core capabilities. Key Iranian cultivars including aromatic Tarom Molaii and high-yielding Khazar and Neda were transformed with genes of interest. Early foundational work at NIGEB established transformation protocols for Iranian rice cultivars.^18^ Foundational work by Kiani et al. demonstrated stable integration of the *cry1Ab* gene via Southern blot analysis, confirming single-copy insertion in multiple independent transgenic events. Mendelian segregation ratios (3:1) were observed in T2 generations, indicating predictable inheritance.^14^ However, the stability evaluation was limited to two generations under greenhouse conditions; multi-generational field data under real farming conditions have not been published.

### Phase II: Trait Pyramiding and Controlled Environment Evaluation (Early 2010s)

Building on stable transgenic events, researchers advanced to stacking multiple traits. A study by Kiani et al. pyramided the cry1Ab insect-resistance gene with the recessive *fgr* gene conferring fragrance, using marker-assisted selection (MAS) to identify progeny carrying both transgenes. The study confirmed successful pyramiding via PCR and demonstrated that fragrance quality (measured by 2-acetyl-1-pyrroline content) was retained in pyramided lines.^15^ These evaluations were conducted under controlled greenhouse conditions; field-level confirmation of the stacked traits has not yet been published.

### Phase III: Confined Field Trials–Agronomic and Environmental Validation (Mid-2010s Onward)

The most critical phase involved multi-location CFTs conducted under strict biosafety protocols. Global meta-analyses confirm that Bt crops generally reduce non-target organism impacts compared to broad-spectrum insecticides.^19^ LCA methodologies for GM crops have been validated in European sugar beet systems.^20^ Baseline energy consumption patterns in Iranian paddy fields provide context for LCA comparisons.^21^ Farm size significantly influences energy efficiency and carbon footprint in Iranian crop production.^22^ Greenhouse vegetable production systems provide comparative data for optimizing energy use.^23^ A comprehensive 2016 trial across three regions (Rasht in Gilan province, Sari and Amol in Mazandaran province) compared several *Bt* rice lines (KHT2, KHT3, KHT4) with their non-transgenic parents. Results showed that *Bt* lines exhibited complete protection against stem borer, with 0% “white heads” compared to 15–20% in susceptible controls. This translated to a yield increase of 18–25% in paddy yield.^16^

Complementing agronomic data, a life cycle assessment (LCA) study of the same trial systems reported that *Bt* rice cultivation, due to drastic reduction (often to zero) in insecticide applications, resulted in lower energy consumption (~25%), reduced global warming potential (~20%), and diminished aquatic toxicity compared to conventional cultivation. The LCA employed a cradle-to-farm-gate system boundary, with a functional unit of one ton of paddy rice, and followed ISO 14040/14044 guidelines.^17^

LCA methodological context: The reported percentage reductions are derived from the specific system boundary and functional unit defined by Dastan et al. (2019). These figures are not directly generalizable to all rice production systems in Iran but provide comparative evidence within the defined scope of the study.

Grain quality analyses confirmed that transgenic lines retained essential characteristics (amylose content 18–22%, gel consistency 80–100 mm, gelatinization temperature intermediate) of their parental cultivars.^16^ However, sensory evaluation or consumer preference studies have not been conducted; laboratory quality metrics alone do not guarantee market acceptability.

## International and Regional Perspectives

To contextualize Iran’s progress, it is useful to examine GM rice research and deployment in other Asian countries:

*China*: Has conducted extensive field trials of *Bt* rice since the 2000s, with studies showing significant pesticide reduction and yield benefits. However, commercial release has been delayed due to regulatory and public acceptance concerns, though recent policy signals suggest potential approval.^24,25^

*India: Bt* cotton is widely adopted, but *Bt* rice remains in research phases despite demonstrated efficacy against stem borer. Regulatory hurdles and activist opposition have prevented commercial release.^26^

*Pakistan*: Research on GM rice (primarily *Bt* and abiotic stress tolerance) is ongoing, with confined field trials reported, but no commercial cultivation to date.

*Bangladesh*: Bangladesh represents the most successful deployment model in South Asia, with Bt brinjal serving as a precedent for potential Bt rice approval.^27^

Recent comparative analyses suggest that regulatory harmonization and public-private partnerships are key differentiators between countries that have successfully deployed GM crops and those that have not^28,29^ (see: https://doi.org/10.1080/21645698.2019.1680241; https://doi.org/10.3389/fsufs.2024.1210385).

Furthermore, trust in GM technology is shaped by trust determinants within a risk/benefit framework, where food technology neophobia plays a contingent role in consumer acceptance.^30^ Post-COVID-19 analyses indicate that consumption value significantly influences consumer willingness to consume GM food, highlighting the need for targeted communication strategies.^31^

## The Policy Quagmire

Iran’s GM rice remains confined to research plots despite compelling evidence. The primary bottleneck is a nonfunctional governance system. The “National Biosafety Law” (2009) was a legislative milestone intended to provide oversight.^32^ However, as documented by Taebi and Karami, its essential implementing regulations and detailed procedural guidelines remain undeveloped. This has created a regulatory vacuum where no government entity–whether the Department of Environment, Ministry of Agriculture, or Ministry of Health–possesses a clear legal mandate, standardized protocols, or defined timelines for reviewing dossier applications or granting approvals for environmental release.^33^

The regulatory stagnation is underpinned by fragmented political decision-making. Investment in agricultural R&D correlates with productivity gains in Iranian agriculture.^34^ There is a palpable absence of high-level political consensus. In this vacuum, a perceived “precautionary principle” has morphed into indefinite risk aversion, where the socio-political risks of making a decision are deemed higher than the risks of inaction.^35^

## Lessons from Bangladesh, the European Union, and Asian Peers

The global landscape offers instructive contrasts:

*European Union (EU)*: The EU’s stringent, politicized approach has resulted in a de facto moratorium on most GM crop cultivation, despite positive scientific opinions from its food safety agency.^36^ Iran shares the EU’s outcome (no cultivation) but lacks its structured regulatory process.

*Bangladesh*: As discussed in Section 5, Bangladesh represents the most successful deployment model in South Asia, with Bt brinjal serving as a precedent for potential Bt rice approval.^27^ Developing countries face unique challenges in balancing GM adoption with organic production systems.^37^

*China and India*: Both countries have conducted extensive GM rice research but have not yet approved commercial cultivation. In China, bureaucratic fragmentation and public opposition have delayed release despite strong scientific evidence.^25^ In India, activist opposition and regulatory caution have prevented Bt rice approval, though Bt cotton is widely adopted.^26^ The US regulatory system offers a product-based model distinct from process-triggered approaches.^38^ Biofortification strategies such as Golden Rice demonstrate the potential of GM technology to address micronutrient deficiencies.^39^

## The Social Dimension

Technical and regulatory hurdles are compounded by a challenging socio-perceptual landscape. A survey of 600 Tehran residents (Ghasemi et al.) reported that 68% of respondents expressed opposition to GM foods, with lack of trust in regulatory oversight cited as the primary factor. The sample was urban and not representative of rural farming populations.^40^

Content analyses of Iranian media from 2015–2020 (Karami & Taebi) examined 450 news articles across 12 outlets, finding that 73% of coverage employed alarmist frames (“biological contamination,” “unknown dangers”), while only 12% included statements from Iranian scientists involved in GM research. Agricultural news platforms provided more balanced coverage, but their audience reach is limited compared to mainstream media.^41^

This creates a feedback loop where public uncertainty justifies political caution, which in turn starves public discourse of authoritative information.

*Reframing strategy*: The narrative should pivot from a defensive discussion of “GMO risks” to a proactive promotion of “public-sector innovation for sustainable agriculture,” highlighting farmer-centric benefits (yield stability, reduced pesticide exposure), consumer-centric benefits (preserved aroma, reduced residues), and national interest benefits (food sovereignty, reduced emissions).^16,17,42–44^ Engaging trusted intermediaries–farmer unions, agricultural scientists, and religious scholars–is critical for credible message dissemination.^45^

## Policy Drivers and the Fate of Biotech Innovation in Iran

The contrasting trajectories of three major biotechnological developments in Iran provide valuable insight into the country’s technology governance framework. While stem cell research flourished under the explicit endorsement of both religious authorities and political leadership, thereby enabling Iran to emerge as a regional leader in the field, mRNA-based COVID-19 vaccines faced import restrictions. These restrictions were justified on the basis of the Islamic juridical principle of “la zarar” (the prohibition of harm), together with concerns regarding unresolved long-term safety data at the time. The case of genetically modified (GM) rice provides a third illustration: despite having successfully passed scientific assessment and regulatory review, its commercialization was ultimately suspended following shifts in national policy priorities. Taken together, these cases suggest that the differing outcomes are not arbitrary but instead reflect a coherent decision-making logic in which the adoption or rejection of a given biotechnology is shaped by a tripartite calculus of national interest (“maslehat”), security considerations, and conformity with foundational religious-legal principles. Rather than indicating a categorical resistance to modern science, this pattern points to a precautionary and context-sensitive approach to technology governance that seeks to balance scientific advancement with broader ethical, political, and strategic considerations while preventing the premature commercialization of technologies perceived as having unresolved implications for national welfare or public safety.

## Synthesis, Discussion, and a Proposed Pathway Forward

The Iranian GM rice saga presents a coherent but stalled innovation journey. The scientific pipeline has produced compelling evidence, but several limitations should be acknowledged: (1) most agronomic data derive from single-season CFTs rather than multi-year commercial-scale cultivation; (2) LCA results are modeled from trial data, not verified on commercial farms; (3) long-term environmental monitoring frameworks have not been developed; (4) compositional equivalence and non-target organism studies are limited. Commercial deployment would normally require these additional steps, including post-release stewardship systems.

The continued delay represents a significant socio-economic opportunity cost, denying farmers a risk management tool and perpetuating unnecessary pesticide use. Opposition to beneficial GM technologies such as Golden Rice carries significant economic costs.^46^ Critical reviews acknowledge both promises and problems of GM foods, necessitating balanced assessment.^47^ Investment in agricultural R&D correlates with productivity gains in Iranian agriculture.^34^

Proposed pathway:

1. *Regulatory activation*: Complete implementing regulations of the 2009 Biosafety Law, creating a transparent, time-bound approval process.
2. *Strategic communication initiative*: Launch a long-term public campaign centered on “National GM Rice,” leveraging data from^16,17^ and religious endorsements.^45^
3. *Pilot-scale managed deployment*: Initiate a limited, closely monitored commercial release in a selected district to generate real-world data on socio-economic impact and strengthen stewardship capacity. This is presented as a high-level policy concept requiring detailed operational design (e.g., selection criteria for districts, monitoring protocols, stakeholder engagement mechanisms) before implementation.

## Conclusion

Iran stands at an agricultural crossroads. Its scientists have delivered a technically sound, potentially transformative tool for sustainable rice production. The past decade of field research has answered many primary agronomic, environmental, and safety questions within the context of confined trials. However, the evidence base has limits: multi-year field data, compositional equivalence studies, and long-term environmental monitoring are not yet available. These are normal steps in a commercial deployment pathway, not fundamental safety concerns.

The remaining questions are political and ethical: Does Iran possess the governance capacity and societal will to translate its own scientific investment into tangible national benefit? The journey of GM rice from laboratory glassware to farmers’ fields is no longer solely a scientific challenge–it is a test of Iran’s ability to manage technological innovation at the intersection of science, policy, and society. The time for decision is overdue.

## Data Availability

No primary datasets were generated for this review article. The work is based on analysis and synthesis of previously published studies, all of which are cited in the reference list.
